# Shape Deformation in Ion Beam Irradiated Colloidal Monolayers: An AFM Investigation

**DOI:** 10.3390/nano10030453

**Published:** 2020-03-03

**Authors:** Valeria Lotito, Marko Karlušić, Milko Jakšić, Kristina Tomić Luketić, Ulrich Müller, Tomaso Zambelli, Stjepko Fazinić

**Affiliations:** 1 Ruđer Bošković Institute, Bijenička cesta 54, 10000 Zagreb, Croatia; marko.karlusic@irb.hr (M.K.); milko.jaksic@irb.hr (M.J.); kristina.tomic@irb.hr (K.T.L.); 2 Laboratory of Biosensors and Bioelectronics, Institute for Biomedical Engineering, ETH Zurich, Gloriastrasse 35, 8092 Zurich, Switzerland; zambelli@biomed.ee.ethz.ch; 3 Nanoscale Materials Science, Empa—Swiss Federal Laboratories for Materials Science and Technology, 8600 Dübendorf, Switzerland; ulrich.mueller@empa.ch

**Keywords:** colloidal particles, colloidal monolayers, polymer particles, air/water interface self-assembly, ion beam modification of materials, particle deformation, atomic force microscopy (AFM) data analysis

## Abstract

Self-assembly of colloidal monolayers represents a prominent approach to the fabrication of nanostructures. The modification of the shape of colloidal particles is essential in order to enrich the variety of attainable patterns which would be limited by the typical assembly of spherical particles in a hexagonal arrangement. Polymer particles are particularly promising in this sense. In this article, we investigate the deformation of closely-packed polystyrene particles under MeV oxygen ion irradiation at normal incidence using atomic force microscopy (AFM). By developing a procedure based on the fitting of particle topography with quadrics, we reveal a scenario of deformation more complex than the one observed in previous studies for silica particles, where several phenomena, including ion hammering, sputtering, chemical modifications, can intervene in determining the final shape due to the specific irradiation conditions. In particular, deformation into an ellipsoidal shape is accompanied by shrinkage and polymer redistribution with the presence of necks between particles for increasing ion fluence. In addition to casting light on particle irradiation in a regime not yet explored, we present an effective method for the characterization of the colloidal particle morphology which can be applied to describe and understand particle deformation in other regimes of irradiation or with different techniques.

## 1. Introduction

Self-assembly of colloidal particle monolayers has emerged as an attractive bottom-up approach to tailor nanostructures and materials with specific properties due to the lower cost and time burden in comparison with top-down approaches based on serial nanofabrication techniques such as electron beam lithography, with the potential for large area and high scale fabrication. Applications range from optoelectronics to chemical and biological sensing, from surface engineering to light harvesting and environmental remediation [1,2]. 

Typically, spherical colloidal particles arranged in hexagonal order are used as sacrificial material as a template for etching or deposition of material in the so-called nanosphere lithography or colloidal lithography process and removed for the creation of the final structure [1,3,4,5,6]. In other cases, colloidal particles can be incorporated in the final structure, as occurs, for instance, in metal film on nanosphere structures employed in surface enhanced Raman scattering [4,5] or in microlenses based on colloidal particles [7].

Among the colloidal materials more commonly employed for nanostructure fabrication based on self-assembly, silica and polymer particles can be numbered. In particular, polymer colloids, for instance polystyrene particles, have emerged as a widespread building block as they exhibit several advantages in terms of choice of chemical composition and functionalization, size, cost, possibility to modify their shape [8].

Independently of the specific application and of its use as sacrificial material or as integral part of the final device, the original monolayer usually consists of hexagonally closely packed spherical particles, which would impose limits on the final attainable morphology. In order to enrich the variety of structures achievable with colloidal monolayers, techniques aiming at modifying the shape and/or the size of colloidal particles have been developed.

One of the most widespread methods is reactive ion etching (RIE), mainly used to shrink colloidal particles so as to turn a closely packed arrangement into a non-closely packed pattern [3,9,10,11]. Using RIE-reduced particles as a mask for metal deposition, one can obtain, for instance, a metal mesh with circular holes, whereas, with the originally closely-packed arrangement, only nearly triangular islands (corresponding to the interstices between closely-packed patterns) would be feasible [4,5]. Other approaches, such as thermal annealing and solvent annealing, can be adopted to tune the shape and size of the interstices between particles [12,13]. 

Ion beam irradiation has also been pursued as a viable route to induce colloidal particle deformation. In particular, several studies conducted on isolated silica particles have demonstrated the deformation of the originally spherical shape into an ellipsoidal shape. This means that, in a reference system for which the axes of the ellipsoid are aligned along the x, y, z axes, the particle surface, originally described for a particle of diameter d by an equation of the form $x^{2}+y^{2}+z^{2}={\left(\frac{d}{2}\right)}^{2}$, is converted into an ellipsoidal shape ${\left(\frac{x}{a}\right)}^{2}+{\left(\frac{y}{b}\right)}^{2}+{\left(\frac{z}{c}\right)}^{2}=1$ where a, b, c are the semi-axes of the ellipsoids and, more precisely, into a spheroidal shape (i.e., with two equal semi-axes, for instance a=b). More specifically, it has been shown that, upon irradiation with an ion beam in the MeV energy range, colloidal particles tend to expand in the plane orthogonal to the ion beam and to shrink in the direction parallel to the ion beam leading to an oblate ellipsoidal shape (for instance with a=b>c, if the ion beam is aligned along z) [14,15,16]. Such shape deformation has been explained in the light of the so-called “ion hammering effect”, which takes its name after the fact that the ion beam acts like a hammer, leading to the shrinkage of the target material in the direction parallel to the ion beam and its dilatation in the direction orthogonal to the ion beam [17]. Hence, by playing with the angle of incidence of the ion beam $\theta_{ion}$ with respect to the normal to the target substrate, one can get ellipsoids with different orientations with respect to the substrate; in addition, subsequent irradiations can be carried out at different angles of incidence of the ion beam to get different shapes; for example, the combined effect of two successive orthogonal irradiations can lead to a prolate ellipsoidal shape (for instance, with a=b<c) [14].

The interaction of the ion beam with the target material is a complex phenomenon, that can be described as a sequence of collisions, which can result in elastic interactions with target atoms (nuclear interactions) and inelastic interactions with electrons (electronic interactions) [18,19]. The total energy transferred to the target atom is the sum of a nuclear and an electronic component, whose relative weight depends on the projectile/target mass ratio and on the ion energy [18,19]. Roughly speaking, a charged particle penetrating into a solid target progressively loses its energy E through the interactions with the host matrix; the total loss per unit length called stopping power ${\left(\frac{\partial E}{\partial x}\right)}_{total}$ can be written as [17,19,20,21,22]:

(1) $${\left(\frac{\partial E}{\partial x}\right)}_{total}=S_{n}\left(E\right)+S_{e}\left(E\right)={\left(\frac{\partial E}{\partial x}\right)}_{nuclear}+{\left(\frac{\partial E}{\partial x}\right)}_{electronic}$$

where:

- $S_{n}\left(E\right)={\left(\frac{\partial E}{\partial x}\right)}_{nuclear}$ is the nuclear stopping power due to elastic nuclear collisions dominant for low energies *E* of the impinging ion;
- $S_{e}\left(E\right)={\left(\frac{\partial E}{\partial x}\right)}_{electronic}$ is the electronic stopping power due to inelastic electronic collisions involving the incident ion and substrate electrons dominant for medium/high energies *E* of the impinging ion.

Hence, the ratio $S_{e}\left(E\right)/S_{n}\left(E\right)$ increases for ion energies increasing from the keV to the MeV range.

As mentioned, most of the studies have focused on silica particles irradiated with different types of ions impinging with an energy such that electronic stopping power is dominant and usually with an incidence angle of the ion beam $\theta_{ion}=45{}^{\circ}$. The effect of several parameters has been considered, for instance of the ion fluence or areal density $N_{I}$ (i.e., the number of ions impinging on the target material per unit area) [14,15,23,24,25], electronic energy loss controlled either via the ion energy E for the same ion or via the type of ion at the same energy E [14,15,16,24,26,27], angle of incidence [25], temperature [24,28] and particle size [14,23,29]. Generally speaking, when all the other conditions are the same, deformation increases with $S_{e}$ and with fluence, while it decreases with temperature. Some results are still controversial, for example concerning the existence or not of a threshold value of $S_{e}$ for deformation [15,16,23]. In previous works, the quantitative characterization of the deformation has been performed by evaluating transverse and longitudinal axes with scanning electron microscopy (SEM). The biaxial expansion in the plane perpendicular to the ion beam direction and the uniaxial contraction along the direction of the ion beam can be estimated by properly choosing the angle of observation of the deformed particles with respect to the angle of irradiation during SEM measurements [14,15,16,23,25,26,27,29,30,31,32].

In addition to being focused on silica, such studies have considered mainly the deformation of nearly isolated particles. However, irradiation-induced deformation of closely-packed monolayers is of major interest for the purpose of the use of colloidal self-assembly for the fabrication of nanostructures. Different behaviours are expected if colloidal particles are isolated or closely-packed as, in the latter case, free deformation of a particle is hindered by its neighbours resulting in mechanical stresses not contemplated in the model of mere ion hammering of a colloidal particle [33]. A visual inspection of SEM images has suggested an expansion still occurring perpendicularly to the ion direction, yet hampered by the presence of the neighbouring particles. In the case of incidence normal to the substrate ($\theta_{ion}=0{}^{\circ}$), the expansion in the plane orthogonal to the ion beam and the contraction parallel to the ion beam leads to a reduction of the size of the interstices between particles and to sliding of the colloids over the substrate, with an increase in the inter-particle distance and buckling of particles over large domains due to large in-plane stresses [34]. For $\theta_{ion}=45{}^{\circ}$, a structure consisting of partially overlapping rows of particles, reminiscent of shingles or roof tiles, has been observed due to the fact that particles are free to expand in one direction perpendicular to the ion beam and constrained in the other direction due to the presence of neighbouring particles [24]. Only the size of the interstices upon increasing fluence has been estimated for a quantitative evaluation of the deformation for closely-packed arrangements [34]. Other studies on closely-packed silica particles have been carried out only in the keV regime [35,36,37,38]: different phenomena have been observed according to the specific experimental conditions, in particular particle reorganization due to the combined effect of charging/discharging and Coulomb repulsion, ion irradiation-induced viscous flow and surface melting due to heating and, in some cases, particle shaping via sputtering, origination of pronounced menisci at the contact points of the spheres, broadening of grain boundaries and interlinking of particles at contact points until complete coalescence.

Only few studies have been devoted to polymer particles. Studies on closely-packed assemblies have been conducted with light keV ions, a regime in which, differently from the aforementioned studies, the contribution of nuclear stopping is non-negligible and even comparable to that of electronic stopping [39,40]. Broadening of grain boundaries due to ion induced charging and interlinking of particles at contact points associated to sintering have been observed [39]. Coalescence and neck formation have been detected under keV ion irradiation also in [40]. We point out, however, that this keV regime is fundamentally different from the MeV regime. MeV ion irradiation of sparse polystyrene assemblies at $\theta_{ion}=45{}^{\circ}$ has revealed a deformation into ellipsoidal shapes, nonetheless with a simultaneous volume shrinkage not found in silica particles [32]. However, for the irradiation conditions of that work (gold ion beams in the energy range 2-10 MeV), electronic and nuclear stopping are of comparable values. Therefore, the estimated threshold for ion hammering in polystyrene at 1.25 $\frac{\mathrm{keV}}{\mathrm{nm}}$ contains both nuclear and electronic stopping contributions that have different efficiencies in materials modification [41,42,43]. Other studies on polymers have been carried out at even higher energies, but on polymer films [44].

In this study, we investigate the deformation of closely-packed polystyrene assemblies under MeV oxygen ion irradiation at $\theta_{ion}=0{}^{\circ}$. In previous works, the effects of ion irradiation have been characterized by SEM, which, however, requires proper selection of the angle of observation for a correct analysis and is not practical to scrutinize shape deformation in closely-packed arrangements. Hence, instead of using SEM for the characterization of ion-irradiated particles, we resort to atomic force microscopy (AFM) to characterize particle deformation by surface fitting. In this way, first of all, we provide a characterization of the effects of ion irradiation in a regime not yet studied for polymer particles, which is of interest to understand particle deformation for practical applications of colloidal self-assembly, but also to get insight into ion irradiation mechanisms. In addition, we outline an investigation approach of colloidal deformation alternative to SEM analysis which is of potential interest for the characterization of colloidal deformation under different conditions and with diverse techniques.

## 2. Materials and Methods 

### 2.1. Self-Assembly of Colloidal Monolayers

Polystyrene (PS) particles of nominal diameters equal to d=300 nm have been purchased from Thermo Scientific (5000 Series Polymer particles packaged as aqueous suspensions at 10 wt % solids; coefficient of variation CV < 3%, Waltham, MA, USA). 

Interfacial self-assembly has been used for the fabrication of colloidal monolayers due to its effectiveness and versatility for the assembly of very diverse materials and has been widely investigated theoretically [1,3,45,46,47,48,49,50,51,52,53,54,55,56]. In particular, air/water interface self-assembly based on surface confinement and water discharge has been adopted [45]. In a quick implementation, a Petri dish is filled with water and a small amount of surfactant (0.1 mM sodium dodecyl sulphate); a nitrile butadiene rubber ring is put on the water surface; particles are dispensed to the air/water interface using a tilted glass slide; the colloidal monolayer thus formed at the interface is transferred to a substrate placed at the bottom of the Petri dish before particle injection by taking water out of the Petri dish.

### 2.2. Ion Beam Irradiation of Colloidal Monolayers

Ion beam irradiations have been performed at the Ruđer Bošković Institute (RBI) Tandem accelerator facility using 1 MV Tandetron accelerator. Negative oxygen ions produced in the source of negative ions by cesium sputtering (SNICS sputtering ion source) were accelerated and directed as 1 MeV $O^{2+}$ ions into the vacuum chamber where colloidal monolayers were positioned on the sample holder equipped with the goniometer with 5 degrees of freedom of movement, including *x*, *y*, *z* translation and azimuth/tilt rotations with 0.01° precision [57]. The ion beam current was measured before and after irradiations using a Faraday cup installed at the sample holder and indirectly controlled by measuring the ion beam current from the sample holder during irradiation. 

In brief, colloidal monolayers have been irradiated with oxygen ($O^{2+}$) ions with energy E=1 MeV and ion beam incidence angle $\theta_{ion}=0{}^{\circ}$. For polystyrene irradiated in such conditions, one has electronic stopping $S_{e}=0.6635\;\frac{\mathrm{keV}}{\mathrm{nm}}$, nuclear stopping $S_{n}=0.0119\;\frac{\mathrm{keV}}{\mathrm{nm}}$ and projected range $R_{ion}=2.22\;\mu m$ according to SRIM-2013 [58,59]. Hence, the maximum energy loss for 1 MeV $O^{2+}$ ions passing through a 300 nm PS particle is approximately 200 keV. After crossing a whole particle diameter, electronic stopping will decrease down to $S_{e}=0.5825\;\frac{\mathrm{keV}}{\mathrm{nm}}$ and nuclear stopping will increase up to $S_{n}=0.0142\;\frac{\mathrm{keV}}{\mathrm{nm}}$. Therefore, the interaction of the 1 MeV oxygen beam with PS is dominated by electronic stopping power which remains fairly constant when passing through the PS particles.

Irradiations at variable fluence $N_{I}$ have been carried out by controlling the irradiation time using the following relationships:

(2) $$N_{I}=\phi_{ion}t$$

with $\phi_{ion}$ being the ion flux (number of ions per unit area and time) equal to

(3) $$\phi_{ion}=\frac{I_{ion}}{qA}$$

where $I_{ion}$ is the ion current, A the area of irradiation, q=n·e is the total charge carried by the ion, with n being the charge state and e the elemental charge unit. Replacing Equation (3) in Equation (2), we get:

(4) $$N_{I}=\frac{tI_{ion}}{neA}$$

Hence, for a given type of ion and known $I_{ion}$ and A, $N_{I}$ can be varied by changing the irradiation time. 

### 2.3. AFM Image Acquisition and Analysis

A Bruker Ikon operated in tapping mode has been used for AFM measurements using TESPA-V2 pyramidal silicon probes. ASCII files, generated with Bruker NanoScope Analysis software (version 1.90), have been imported in Matlab for further analysis. Tilt subtraction has been performed to correct for possible substrate tilt during AFM measurements before surface fitting with quadrics.

## 3. Results and Discussion

Preliminary calculations have been performed using SRIM software to estimate the electronic stopping power $S_{e}$ and the nuclear stopping power $S_{n}$ for oxygen ions impinging on polystyrene at the irradiation ion energy E=1 MeV. In such conditions, we have $S_{e}=0.5825-0.6635\;\frac{\mathrm{keV}}{\mathrm{nm}}$ and $S_{n}=0.0119-0.0142\;\frac{\mathrm{keV}}{\mathrm{nm}}$ as previously explained, meaning that $S_{e}\gg S_{n}$. This ensures that we are in a regime in which ion/target interactions are dominated by inelastic electronic collisions. In addition, the so-called projected range or ion penetration depth $R_{ion}$ along the direction of incidence is equal to 2.22 μm, greater than the particle diameter *d*.

**Figure 1:**
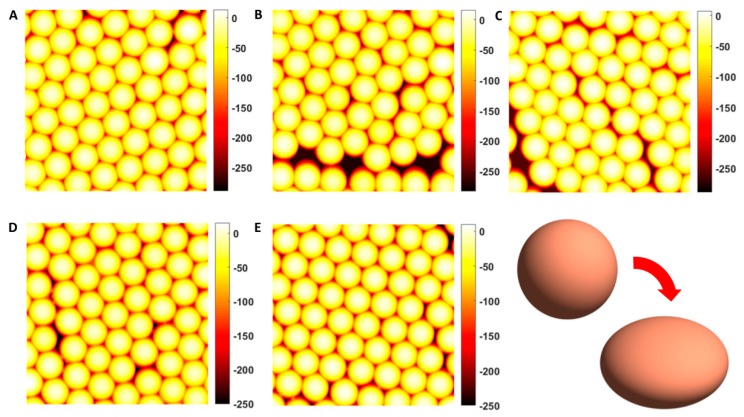
Examples of AFM micrographs for increasing ion fluence. The size of each micrograph is 2 µm × 2 µm. The fluence values for each of the plots correspond to those reported in Figure 2 (the different fluence values are indicated with letters **A**–**E**). Sketch of the deformation of a sphere into an oblate ellipsoid.

**Figure 2:**
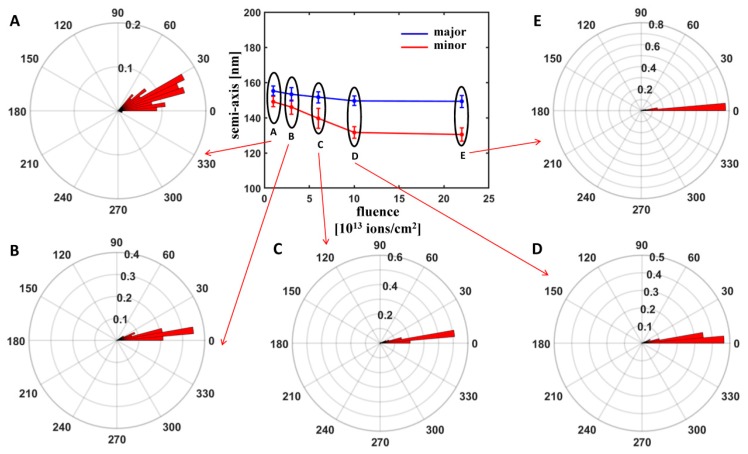
Characterization of the deformation of irradiated colloidal particles for variable fluence by fitting with quadratic surfaces: major/minor semi-axis length and polar histograms showing the probability distribution of the orientation of the angle *α* formed between the minor semi-axis and the normal to the substrate surface (the different fluence values are indicated with letters **A**–**E**).

Figure 1 shows AFM images of particles for variable fluence $N_{I}$ (the different fluence values are indicated with letters A–E as reported in Figure 2). A preliminary visual inspection apparently suggests a preservation of a nearly circular shape in the projection, but reveals a progressive decrease in the total height range of the image and the appearance of a sort of necks between neighbouring particles which becomes more evident for increasing $N_{I}$.

Since we are in a regime for which deformation into an ellipsoidal shape has been observed in previous studies on isolated silica particles, we have performed a fit with a quadratic surface (i.e., a second-order algebraic surface, also referred to as quadric) for our particles, limiting the analysis to the upper cap of the particles accessible via AFM measurements and to the portion of particle surface not affected by the presence of the neighbouring particles to check whether such approximation is valid also for closely-packed polystyrene particles. We observe that the condition $R_{ion}>d$ should ensure that the particles are fully irradiated; we remind that, in isolated silica particles, it has been observed that, for $R_{ion}<d$, deformation is restricted to the sole irradiated region [15,16,28].

Table 1 reports the general algebraic quadratic equation in three variables. In order to discriminate between the different quadrics, one considers the rank of the matrices e and E, the determinant of the matrix E and the sign of the eigenvalues of e and E [60,61,62,63]; for instance, the characteristic numbers $\lambda_{1}$, $\lambda_{2}$ and $\lambda_{3}$ of e are defined as the roots of:

(5) $$\left|\begin{array}{ccc} a-\lambda & h & g\\ h & b-\lambda & f\\ g & f & c-\lambda \end{array}\right|=0$$

There exist 17 types of surfaces of this type [61,62,63]. For this analysis, we are first interested in determining whether irradiated particles can be fitted with a real ellipsoid, whose properties are reported in Table 1; more details can be found in specialised literature [60,61,62,63]. Table 1 reports also other two quadratic surfaces that will be discussed later.

We observe that the equation in Table 1 can describe an ellipsoid with arbitrary orientation of the axes with respect to the reference system and with arbitrary length of the semi-axes (which could be all different in the most general case of a tri-axial ellipsoid). This makes the procedure of surface fitting of AFM data more general and comprehensive in comparison with the analysis based on SEM images because it does not require any assumption on the mechanisms of deformation and, consequently, on the semi-axes lengths and orientation. In fact, during SEM measurements, the biaxial expansion and the uniaxial contraction have been estimated by properly choosing the angle of observation of the deformed particles with respect to the angle of irradiation, assuming that the contraction occurs along the direction of irradiation and the dilation in the orthogonal plane. However, deviations in the actual orientation of the ellipsoids have been observed in real experiments. For instance, for isolated silica particles irradiated at $\theta_{ion}=45{}^{\circ}$, the inclination of the plane of expansion with respect to the substrate surface has been shown to be smaller than the expected value for increasing fluence due to the gradual variation in the geometry of the contact area between the colloid and the substrate because of particle expansion [15,28]. In addition, a deviation not only in the orientation of the ellipsoid but also in the relative value of the semi-axes (i.e., in the model of uniaxial contraction and biaxial expansion) has been theoretically predicted for irradiations with very high fluences and $\theta_{ion}=45{}^{\circ}$, for which the three semi-axes in the ellipsoid are expected to be all different [33]. Therefore, assumptions on semi-axes lengths and orientation cannot be easily made a priori in the most general case. Hence, this approach based on quadric fitting of AFM data can be applied to determine the characteristics of the ellipsoidal deformation without any prior hypothesis and circumventing the need for a cumbersome choice of the observation angle and a selection of proper visualization conditions, which becomes more complex especially if particles are closely-packed.

For all the analysed fluences, particle surfaces could be fitted with ellipsoids characterized by one semi-axis lower than the other two approximately equal semi-axes. This could be compatible with a scenario of deformation into an oblate ellipsoidal shape (a=b>c). In order to understand if such a deformation can be explained in terms of the ion hammering effect dominant in isolated silica particles in the regime of predominance of electronic stopping power, we have computed the angle α formed between the minor semi-axis and the normal to the substrate surface. Figure 2 reports the histogram plot of this angle for variable fluence, together with the average length of the semi-minor and semi-major axes. 

We observe that the angle α is very close to zero; as irradiations have been carried out at $\theta_{ion}=0{}^{\circ}$, this confirms the deformation into an oblate shape with minor semi-axis along the ion beam direction and major semi-axes in the plane orthogonal to the incidence direction; the larger dispersion of values for the lowest fluence can be explained with the fact that, at such value, the difference between minor and major semi-axes is still negligible, meaning that the surface is still close to a spherical shape. 

Concerning the semi-axes lengths, only for the lowest fluence a slight reduction in the direction of the ion beam irradiation and a slight expansion in the orthogonal plane with respect to the original spherical particle (for which $a\approx b\approx c\approx \frac{d}{2}$) is observed. Upon increase in $N_{I}$, a decrease in both the minor and major semi-axes is detected, more pronounced for the minor semi-axis and less significant for the major semi-axes, implying particle volume loss. This behaviour is different from that found in the case of isolated silica particles for which the decrease in the minor semi-axis is accompanied by an increase in the major semi-axes for raising fluence so as to preserve volume [14,24]. It also departs from the theoretical description of semi-axes evolution within the framework of the ion hammering model, which supposes volume conservation [33].

In order to understand our findings, we should recall that ion/target interaction is a complex phenomenon in which several phenomena occur simultaneously.

For instance, besides the ion hammering effect, another phenomenon of potential interest in determining particle deformation is ion sputtering, consisting in erosion of the surface of the target material under ion bombardment, whereby surface atoms are removed by primary and secondary collisions of the impinging and recoiled atoms with the target atoms; the sputtered material can be an ion or neutral [64]. In previous works, sputtering has been invoked as explanation for volume shrinkage in very different ion irradiation conditions. For instance, in irradiation of closely-packed silica particles with $S_{e}<S_{n}$ and $R_{ion}<d$ [65] sputtering-induced surface erosion has been observed at higher fluences, with a consequent reduction of particle volume, with a fluence-dependent sputtering rate affected by the simultaneous particle flattening and reduction of free surface area for sputtering due to the concomitant ion hammering process. Sputtering of silica particles in similar conditions has been observed also in [36,37,38]. Sputtering is more important especially in lower energy range where $S_{n}$ is significant in comparison with $S_{e}$, a condition met in the aforementioned studies [64], even if sputtering in the electronic regime has also been reported [20,66].

In addition, chemical effects occurring during ion irradiation are also very important. In particular, the energy release of fast ions in polymers, in the range from several keV to MeV, induces deep changes in chemical and physical properties, with breaking and re-arrangement of the original chemical bonds [64,67]. Such variations may entail several effects, ranging from cross-linking between chains (when free dangling ion or radical pairs on neighbouring molecular chains join) and chain scissions, heavy damage leading to properties close to those of hydrogenated amorphous carbon, graphitization, modification of functional groups, destruction of aromaticity, formation of a three-dimensional (3D) compacted network [64]. These effects are dependent on the total ion deposited energy, electronic stopping $S_{e}$ and nuclear stopping $S_{n}$ (which both play a role in the modification of polymer properties differently from what generally occurs in semiconductors and metals), chemical composition and structure of polymers, ion fluence [64,67,68]. For instance, typically, at lower fluences ($\sim 10^{14}\;{\mathrm{cm}}^{-2}$), cross-linking between chains and chain scissions are dominant, while, for increasing higher fluences, heavy damage of the original polymer is originated, with properties approaching those of hydrogenated amorphous carbon ($\geq 10^{15}\;{\mathrm{cm}}^{-2}$) and even grafitization ($\sim 10^{16}\;{\mathrm{cm}}^{-2}$) [64,67,68]. Moreover, various gaseous molecular species are released, e.g., hydrogen and less abundant heavier molecules which are scission fragments [64]. The amount of cross-linking and scission affects mechanical and physico-chemical properties (i.e., mechanical stability, molecular weight distribution, rheology, solvent in-diffusion and solubility) as well as electrical conductivity, charge carrier mobility, electronic density, optical density [64]. Cross-linking and scission can result from both electronic and nuclear energy transfer, even if, generally speaking, nuclear stopping is likely to cause more scission, while electronic stopping induces prevalently cross-linking [41,69].

Of particular interest for volume loss are those chemical modifications that involve either a loss of mass or the formation of a three-dimensional compacted network by densification: the first process may stem from irreversible cleavage of bonds within a molecule, resulting in fragmentation of the molecule and loss of various volatile species; the second process occurs via extensive processes of backbone rearrangement and cross-linking with the formation of chemical bonds between different molecules or different parts of a macromolecule [68]. The radiation damage leading to the escape of volatile chemical elements (degassing) can cause enrichment by carbon (carbonisation) [70]. Concerning polystyrene, both the phenomena have been observed, with the consequent reduction of the thickness of polystyrene films due to increase in polymer density and hydrogen outgassing [71]. Modification of polystyrene molecular structure has been demonstrated under different irradiation conditions [72,73,74,75]. Chemical modification has been proposed as a possible explanation to the volume shrinkage observed in sparse polystyrene particles at $\theta_{ion}=45{}^{\circ}$ [32] and in closely-packed polystyrene particle arrangements in a regime of irradiation in which $S_{n}$ is comparable with $S_{e}$ [39]. Oxygen exposure both during irradiation and after irradiation can also play a role in inducing chemical changes [70,76,77].

**Figure 3:**
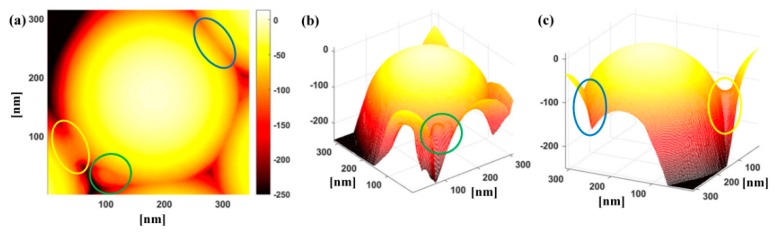
Polymer redistribution occurring during ion irradiation: (**a**) polyhedral-like deformation at the contact area with a neighbouring particle (within the blue line), neck between neighbouring particles (within the yellow line), protrusion due to broken connection (within the green line); (**b**) close-up and three-dimensional view of the protrusion due to broken connection (within the green line); (**c**) close-up and three-dimensional view of the deformation at the contact area with a neighbouring particle (within the blue line) and of a neck between neighbouring particles (within the yellow line).

We point out that, even if volume reduction is possible due to ion irradiation-induced sputtering or chemical modifications, polymer redistribution should also not be neglected. In fact, while the upper cap of the particles in the portion not affected by the neighbouring particles can be fitted with ellipsoids whose semi-axes could indicate an overall volume loss, yet flow of polystyrene towards the interstices between particles and along the line connecting two neighbouring particles can also occur. Figure 3 shows, for instance, a particle whose upper cap preserves an ellipsoidal shape, but that exhibits deformations at the contact points to neighbouring particles or towards the interstices, as indicated with the coloured lines. Below the contact points with neighbouring particles, deformation into nearly polyhedral shape can take place (encircled by the blue line). Such a phenomenon has been suggested as the result of lack of space for further expansion, leading to polymer flowing towards the interstices and progressively closing them as observed in silica particles [34,65]. In addition, we observe the presence of necks or bridges connecting particles (enclosed by the yellow line) and other protrusions (surrounded by the green line) probably ascribable to broken connections. 

Necking in ion-irradiated particles is particularly interesting. So far, it has been reported in ion irradiation in regimes in which $S_{n}$ is comparable with $S_{e}$ in closely-packed arrangements of both silica and polystyrene particles; in silica particles even coalescence and strong particle rearrangement have been observed with major disruption of the original particle shape and arrangement at very high fluences [35,36,37,38,39]. Such phenomena have been explained as the effect of ion beam-induced sintering [35,39].

**Figure 4:**
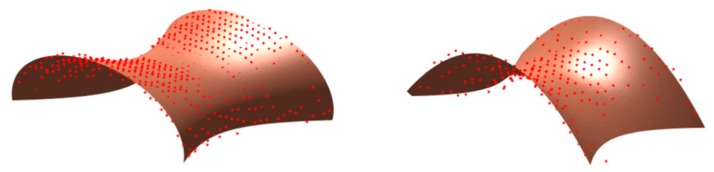
Fits of necks/bridges between neighbouring particles with a one-sheet hyperboloid (**left**) and a hyperbolic paraboloid (**right**).

A visual inspection of the necks would suggest a saddle surface shape. A description of such surfaces can be found in [63]. Figure 4 shows examples of such fits with a hyperbolic paraboloid and a one-sheet hyperboloid. We point out that hyperboloid-like surfaces have been theoretically proposed (together with other curves of similar shape such as the minimal surface known as catenoid) as morphological description of necks occurring during sintering, of capillary bridges between particles and of droplet coalescence [78,79,80,81,82,83], but their assessment is hampered by characterization tools that do not provide a 3D reconstruction. Although a full characterization would require a more extensive study, such fits show how 3D characterization accessible via AFM imaging can provide new insights into the shape and physics behind such necking, in comparison to the sole SEM measurements, through which only the mean width of the projection of such necks has been examined [39]. 

**Figure 5:**
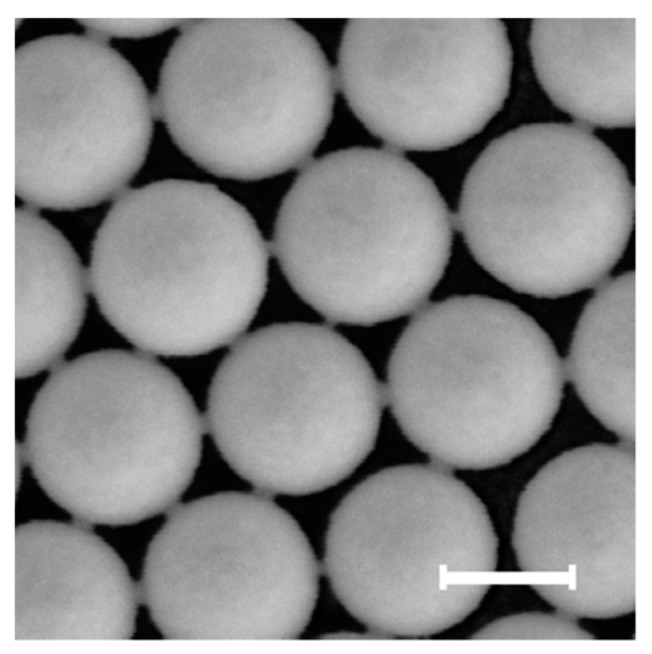
SEM micrograph showing polystyrene particle necking induced by reactive ion etching (scale bar = 200 nm).

It is noteworthy to observe that formation of bridges between particles can be induced also by other particle modification techniques. To discuss this point, we have performed reactive ion etching on particles in mixed oxygen-nitrogen atmosphere as described in [3]. Figure 5 shows necks formed between particles that we treated with reactive ion etching. Such a phenomenon has been often observed with SEM for particles modified by RIE [9,84,85,86,87]. In [84], the effect has been attributed to the transformation of the singular contact point between original particles into extended contacting faces between particles and to plasma-enhanced polymer diffusion at the particle surface due to etching-induced heating at a temperature T higher than the glass transition temperature $T_{g}$ of the particle material and promoted also by the formation of shorter polymer fragments with more efficient diffusion in comparison to the original long polymer strands under oxygen plasma treatment. Neck formation has been observed also under thermal treatment [13,88,89]. 

Getting insight into their shape can hence be of interest in order to investigate mechanisms behind neck formation, validate theoretical models, understand the role of viscous flow and diffusion in the final resulting shape and envisage potential applications of such deformation.

In brief, AFM investigation of irradiated polystyrene particles based on fitting of particle surfaces with quadrics has revealed the deformation of the original spherical shape into oblate ellipsoids with minor axis oriented parallel to the direction of irradiation. This effect is compatible with the ion hammering scenario amply discussed for isolated silica particles, according to which a particle should contract along the direction of the ion irradiation and expand in the orthogonal plane. 

Nonetheless, differently from such studies where particle volume is conserved after deformation, ellipsoid fitting would indicate a simultaneous volume shrinkage for increasing fluence. Such shrinkage could be ascribed to other ion/target interaction phenomena, i.e., sputtering and chemical modifications induced in polymers. Sputtering implying material removal has been previously observed in closely-packed particles irradiated in a regime in which $S_{n}$ is non-negligible in comparison with $S_{e}$, as it is typically dominant in such conditions. Chemical modifications can be originated by both nuclear and electronic stopping and have been demonstrated to induce volume shrinkage in polymers either by irreversible cleavage of bonds and release of volatile species or by extensive backbone rearrangement and cross-linking causing polymer densification.

Although volume reduction is a realistic scenario due to ion irradiation-induced sputtering or chemical modifications, polymer redistribution should also be considered. Polymer protruding out of the particles is observed and, in particular, the presence of necks connecting neighbouring particles. Tentative fits of such necks with saddle surfaces, such as one-sheet hyperboloids have been performed. Similar shapes have been theoretically predicted within the framework of the analysis of phenomena such as particle sintering and droplet coalescence. Necking effects occurring in silica and polystyrene particles in ion irradiation conditions in which $S_{n}$ is non-negligible in comparison with $S_{e}$ have been attributed to ion-induced sintering.

In addition to elucidate polystyrene particle deformation in a regime of irradiation not yet examined, we have outlined an effective method of assessing particle deformation upon ion irradiation. 

So far, the study of ion-induced effects in colloidal particles has been based on the analysis of SEM images. In order to estimate, for instance, deformation into ellipsoids, images at proper angles of observation need to be acquired, which implies hypotheses on the type of deformation occurred and on the physical mechanisms underlying deformation (for example, deformation into an ellipsoid with a certain orientation with respect to the substrate due to ion hammering). Discrepancies with such assumptions have nevertheless been found, which can lead to inaccuracies in the quantitative characterization or impose cumbersome measurement procedures, that become even more complex if particles are closely-packed. Moreover, it is not suitable for the analysis of particles for which a deformation model is not entirely developed. Analysis based on surface fitting of AFM images is more general as it requires fewer assumptions on the actual deformation (for example by fitting the surface generically with a quadric without hypothesis on the spheroid type and its orientation) and can be easily extended to different types of deformation fitting. 

In addition, the availability of 3D data allows a more complete investigation of specific types of deformation. This is the case, for example, of the necking between particles, a phenomenon that is of interest not only for ion-irradiated particles, but also for those subject to different types of treatments such as reactive ion etching or thermal treatment. Analyses based on SEM images rely on the evaluation of neck width; fitting of neck surfaces measured with AFM with different types of curves can provide a more effective comparison with shapes predicted by theoretical models and help to shed light on the physico-chemical mechanisms behind deformation.

Furthermore, the approach could be applied to the description of the morphology of the upper surface of particles deformed in a way that strongly departs from a regime of preservation of point symmetry, as is the case, for instance, of particles subject to ion irradiation in experimental conditions such that $d>R_{ion}$ for which deformation occurs only in the top ion-irradiated portion of the particle [28] or for the biconvex shape deformation frequently observed in case of particles undergoing reactive ion etching [3]. The approach could be inherently useful to assess a wide gamut of deformed particle morphologies provided that the particle size is not smaller or comparable with probe size in order to avoid potential artifacts and could be extended to other scanning probe microscopy techniques providing information on the topography of the upper part of particles, such as scanning near field optical microscopy [90,91,92,93,94].

Further understanding of the mechanisms behind the observed deformation could come from the use of AFM for the evaluation of mechanical properties, which could help to get insight into chemical changes in the particles. In previous works, for instance, AFM analysis of the compressive modulus of isolated polystyrene particles has revealed its dependence on the molar percentage of poly(vinylbenzyl(trimethyl)ammonium chloride) (PVBTA) [95]. Moreover, the determination of the elastic modulus via AFM has been used to assess polymer redistribution effects of colloidal particles under solvent annealing [96]. Hence, the analysis of mechanical properties could help to shed further light on the different mechanisms behind deformation of polystyrene particles upon ion irradiation that we have described to explain the observed morphological variation.

Another interesting outlook for future investigations on deformation of polymer particles would be the analysis of the effect of particle size on ion irradiation via AFM. It has been demonstrated that size influences glass transition temperature and elastic properties of polymer particles due to differences in the arrangement and packing of polymeric chains in the surface and sub-surface regions [97,98,99], which could potentially affect the mechanisms of interaction with ion beams. The irradiation of single-sized colloidal particles of variable size could cast light on the influence of size in such interaction mechanisms. In addition, the simultaneous irradiation of particles of different sizes in binary and, more generally, polinary colloidal assemblies could result in novel colloidal morphologies of potential interest in practical applications of colloidal monolayers.

## 4. Conclusions

We have investigated the morphology of monolayers of closely-packed polystyrene particles irradiated with oxygen ions impinging at normal incidence with respect to the substrate for increasing fluence in a regime in which the electronic stopping power $S_{e}$ is much higher than the nuclear stopping power $S_{n}$, the overall stopping power is below $1\;\frac{\mathrm{keV}}{\mathrm{nm}}$ and the projected range $R_{ion}$ is greater than the particle diameter d. To this end, we have characterized the shape of irradiated particles by AFM and developed a procedure based on fitting with quadrics to examine the deformation for increasing ion fluence. 

The goal of our study was twofold. First of all, we have investigated ion irradiation in a regime not yet studied for polymer particles, which is of interest to understand particle deformation for practical applications of colloidal self-assembly, but also to get insight into ion irradiation mechanisms. In fact, while silica particles have been largely investigated in both isolated and closely-packed arrangements, studies dedicated to polymer particles are less frequent and focused on different irradiation conditions. Second, we have outlined an investigation approach of colloidal particle deformation based on surface fitting applied to AFM images alternative to SEM analysis adopted in previous works on the ion irradiation of particles.

Concerning the first point, we can conclude that the transformation induced by oxygen ion irradiation in closely packed polystyrene particles for $S_{e}\gg S_{n}$ is more complex than the deformation of isolated silica particles in similar conditions. In addition to the deformation into an ellipsoidal shape, volume variation and material redistribution effects related to sputtering, ion-induced chemical modifications and presence of neighbouring particles play a role leading to simultaneous shrinkage and necking. Such effects have been previously observed in polymer particles in a regime in which the nuclear stopping power is non-negligible. Our AFM investigations have cast light on the fact that the morphology of polystyrene particles in this regime is the outcome of multiple concurrent processes.

As to the second point, the procedure based on AFM measurements and surface fitting is more general in comparison to SEM characterization. In fact, SEM characterization requires the acquisition of images at a proper angle of observation and with specific assumptions on the mechanisms of deformation, which, in general, are not known a priori, with consequent possible inaccuracies in shape characterization. In addition, SEM characterization can become complex and cumbersome if particles are closely-packed. By fitting AFM data with quadrics, we could provide a description of shape evolution with fluence without any preliminary assumption on ellipsoid semi-axes length and orientation. In addition, the procedure can be extended to fitting with different types of curves. Hence, the surface fitting approach applied to AFM images of colloidal particles can give new insight into the morphology of colloidal particles deformed by different techniques, help to understand the mechanisms behind deformation and anticipate possible applications.

## Figures and Tables

**Table 1:** General quadratic equation in three variables and definition of the properties satisfied by the quadratic surfaces of interest for particle analysis [60,61,62,63].

$ax^{2}+by^{2}+cz^{2}+2fyz+2gzx+2hxy+2px+2qy+2rz+d=0$			
	Real Ellipsoid	One-Sheet Hyperboloid	Hyperbolic Paraboloid
$\rho_{3}=\mathrm{rank}\;e=\mathrm{rank}\;\left[\begin{array}{ccc} a & h & g\\ h & b & f\\ g & f & c \end{array}\right]$	3	3	2
$\rho_{4}=\mathrm{rank}\;E=\mathrm{rank}\;\left[\begin{array}{cc} \begin{array}{ccc} a & h & g\\ h & b & f\\ g & f & c \end{array} & \begin{array}{c} p\\ q\\ r \end{array}\\ \begin{array}{ccc} p & q & r \end{array} & d \end{array}\right]$	4	4	4
Δ=det E	negative	positive	positive
